# The acceptability of HPV vaginal self-sampling for cervical cancer screening in Latin America: A systematic review

**DOI:** 10.1016/j.puhip.2023.100417

**Published:** 2023-07-31

**Authors:** Luisa Narvaez, Manuela Viviano, Cheryl Dickson, Emilien Jeannot

**Affiliations:** a Institute of Global Health - Faculty of Medicine, Chemin de Mines 9, 1202, Geneva, Switzerland; b Gynecology Division, Department of Obstetrics and Gynecology, Geneva University Hospitals, Boulevard de la Cluse 30, 1205, Geneva, Switzerland; c Community Psychiatric Service, Lausanne University Hospital (CHUV), Lausanne, Switzerland

**Keywords:** Vaginal self-sampling testing, Latin-American women, HPV, Acceptability cervical cancer screening

## Abstract

**Objective:**

This review summarizes women’s acceptability of vaginal self-sampling for cervical cancer screening in Latin America.

**Study design:**

Systematic review

**Method:**

A systematic literature search was performed in PubMed, Web of Science, and Embase regarding the acceptance of HPV vaginal self-sampling by women over 18 years old. Articles were selected for research that was conducted in Latin America and published between January 1st, 1993, and December 31st, 2022.

**Results:**

Fifteen publications were included. Eight publications reported an acceptance of HPV self-sampling as high as 80%, six papers found an acceptance rate between 50 and 80% and only one found an acceptance rate of less than 50%. Based on non-standardized questionnaires, women considered self-sampling more comfortable, easier, and less painful than conventional cytology. The procedure was associated with less embarrassment and a greater sense of privacy.

**Conclusion:**

HPV vaginal-self sampling appears to be an acceptable screening method amongst eligible Latin American women.

## 1 Introduction

Cervical cancer is a malignant tumor of the cervix, the most distal part of the uterus which connects with the vagina [1,2]. It is the fourth most common gynecologic malignancy and the fourth leading cause of cancer death worldwide [3]. Incidence and mortality vary widely with geographic location [4]; recent reports ranked cervical cancer as the third most common neoplasia affecting women in Latin America and in the Caribbean region [3]. In 2020, there were 59,439 estimated new cases of cervical cancer and 31,582 deaths due to this malignancy in this region [5]. Approximately 85% of the new cases and deaths occur in low- and middle-income countries (LMICs) [3,6]. Persistent infection with high-risk types of Human Papillomavirus (hrHPV) -such as 16, 18, 31, 33, 35, 39, 45, 51, 52, 56, 58, 59, 68 [7] - has been identified as the leading risk factor of cervical cancer, being responsible for up to 90% of cases of squamous cell carcinoma, where the hrHPV types 16 and 18 are the most prevalent isolated (70%) of cervical cancer samples worldwide [1,8,9].

Cervical cancer is largely preventable disease due to the highly effective HPV vaccine [4] and secondary prevention measures. Standard secondary measures include Pap smears (cervical cytology), visual inspection with acetic acid (VIA), and Lugol’s iodine, which can detect precursor and early-stage disease [3]. However, access to HPV immunization is insufficient, especially in LMICs [10,11].

The coverage and access to a screening programme are limited outside high-income countries, due to the limited access to health services, paucity of resources, and social, economic, and political issues. In Latin America, several countries have attempted to establish national screening programs without achieving high quality and coverage [12]. In addition, ethnic, religious, and cultural challenges combined with women’s subjective experiences of shame, pain, and discomfort from cervical cancer screening tests result in a reduced number of screening women, which leads to the high incidence of this disease in LMICs, which is why it continues to be an important cause of cancer morbidity and mortality [4,13,14].

A promising strategy to overcome multiple barriers to cervical cancer screening, particularly in low-resource settings, is the Human Papillomavirus (HPV) vaginal self-sampling method. This novel alternative has been developed to decrease cervical cancer mortality worldwide with a considerable impact on decreasing the disease burden and overall health inequalities [15]. Since 2013, the World Health Organization (WHO) has recommended HPV self-sampling as a cost-effective option for initial screening. If the woman desires, she can perform it in the comfort of her own home and send the sample to the health center or laboratory for processing; those screened positive will undergo more extensive testing. HPV DNA Self-sampling test has the potential to reach under screened women, such as those who have never been screened and the ones who do not attend screening regularly [16].

Despite the high disease burden, limited studies have been conducted to evaluate the acceptance of HPV self-testing in Latin America. To our knowledge, no systematic review has been published on this specific subject. The aim of this review is to investigate women’s acceptability of HPV vaginal self-sampling for cervical cancer screening in Latin America, as reported by articles published between January 1st, 1993, and December 31st, 2021. This systematic review will constitute valuable reference materials for epidemiologists, health policymakers, stakeholders, and researchers on cervical cancer to show if this screening method can increase adherence to cervical cancer screening in Latin America. This may be applicable to other LMICs.

## 2 Method

### 2.1 Vaginal HPV self-sampling testing definition

The HPV self-sampling test is a feasible and accurate collection method used by the patient who wishes to know if an HPV infection is present [17]. This process can be carried out alone in private, at home, or at a health facility center. Vaginal self-sampling involves the patient obtaining a kit (a single-use swab or cervical brush and a tube containing a transport medium to collect a cervicovaginal sample) and collecting instructions. The patient gently inserts the swab or brush into the vagina and delicately rotates it for 10 to 30 s to take the sample. After removing the swab, it is transferred into the tube with the transport medium, where the shaft of the swab is broken off and discarded, the tube is sealed and labeled and, finally, is sent to be analyzed at a certified laboratory. The patient receives the results directly at the health facility center, or by telephone by a nurse or doctor from the health center or transmitted by the community health workers. HPV DNA testing identifies the users with a higher risk of developing HPV-related cervical cancer in the future; in the case of positive test results, the women are invited to attend and appropriate health facility for further assessments [18,19].

### 2.2 Literature search strategy

Following PRISMA (Preferred Reporting Items for Systematic Reviews and Meta-Analyses) guidelines, a focused electronic systematic literature search was carried out in PubMed, Web of Science, and Embase for studies conducted in any Latin-American country (Argentina, Bolivia, Brazil, Chile, Colombia, Costa Rica, Cuba, Dominican Republic, Ecuador, El Salvador, Guatemala, Haiti, Honduras, Mexico, Nicaragua, Panama, Paraguay, Peru, Puerto Rico, Uruguay, and Venezuela)

and published between January 1st, 1993, and December 31st, 2022, - 1993 was chosen as the cut-off year because it was the year of the first report on HPV self-sampling [20].

The Keywords used for the research were (HPV[tw] OR “Human Papillomavirus”[tw] OR “Human Papilloma Virus*”[tw] OR “HPV, Human Papilloma Virus*”[tw] OR “Papillomavirus Infections”[Mesh] OR “Papillomavirus Infection*”[tw] OR “Human Papillomavirus Infection*”[tw] OR “HPV Infection*”[tw]) AND (“Vagina”[Mesh] OR “Vaginal”[tw] OR “Cervico-vaginal”[tw] OR “Cervicovaginal”[tw]) AND (“Self-Examination”[Mesh] OR “Self-Examination*”[tw] OR “Self Examination*”[tw] OR Self-sampl*[tw] OR “self sampl*”[tw] OR “Self-collect*”[tw] OR “Self collect*”[tw] OR “Self-test*”[tw] OR “Self test*”[tw] OR “Self-administ*”[tw] OR “Self administ*”[tw] OR “Self-obtained”[tw] OR “Self obtained”[tw] OR “Self-assessment”[tw] OR “Self assessment”[tw]) AND (“Cervical Cancer”[tw] OR “Uterine Cervical Cancer*”[tw] OR “Cancer of the Cervix”[tw] OR “Cancer of Cervix”[tw] OR “Cancer Cervix”[tw] OR “Cancer of the Uterine Cervix”[tw] OR “Cervix neoplasm*”[tw]) AND (“Early Detection of Cancer”[Mesh] OR “Cancer Early Detection”[tw] OR “Cancer Screening”[tw] OR “Screening, Cancer”[tw] OR “Cancer Screening Test*”[tw] OR “Early Diagnosis of Cancer”[tw] OR “Cancer Early Diagnosis”[tw]) AND (“Argentina”[tw] OR “Bolivia”[tw] OR “Brazil”[tw] OR “Chile”[tw] OR “Colombia”[tw] OR “Costa Rica”[tw] OR “Cuba”[tw] OR “Dominican Republic”[tw] OR “Ecuador”[tw] OR “El Salvador”[tw] OR “Guatemala”[tw] OR “Haiti”[tw] OR “Honduras”[tw] OR “Mexico”[tw] OR “Nicaragua”[tw] OR “Paraguay”[tw] OR “Panama”[tw] OR “Peru”[tw] OR “Puerto Rico”[tw] OR “Uruguay”[tw] OR “Venezuela”[tw] OR “Latin America”[Mesh] OR “Hispanic or Latino”[Mesh] OR “Latinas”[tw]) AND (“Women”[Mesh] OR “Female”[Mesh]). Each search strategy was adapted to consider the differences in the controlled vocabulary and the syntax rules.

### 2.3 Study selection criteria

Studies meeting the following inclusion criteria were selected: 1) Studies conducted in Latin American countries; 2) Studies conducted with Latin American women; 3) Studies in women of at least 18 years of age who had completed self-sampling tests; 4) Studies involving pregnant or non-pregnant women, with or without HIV infection and minority ethnicities; 5) Studies measuring the acceptability of vaginal HPV self-sampling test; 6) Studies conducted on primary cervical cancer screening; 7) Studies using a vaginal self-sampling device including swab, brush or tampons; 8) Both quantitative and qualitative studies; 9) Articles reported in English, Spanish, French, and Portuguese due to the linguistic competence of the researcher. Duplicate papers, articles with unclear or lacking methodology, and publications that did not provide sufficient data on the Latin American population were excluded.

### 2.4 Data collection and analysis

Search results were exported to Zotero software, version 5.0. A standardized data abstraction form created on Microsoft Excel, version 16.56, recorded the relevant information for each study: country location, authors, publication year, study design, sample size, population characteristics, intervention, setting, general acceptability, and global experience.

### 2.5 Study quality assessment

Table 1 presents the study quality criteria based on the National Heart, Lung and Blood Institute (NHLBI) guidelines [21]. One researcher assessed the articles according to the aforementioned quality criteria and observed that out of the fifteen included manuscripts, only one was rated as high quality. Seven papers were classified as moderate quality, and an additional seven were considered low to moderate quality. Notably, none of the publications met the criteria for being classified as low or moderate to high quality.

**Table 1:**
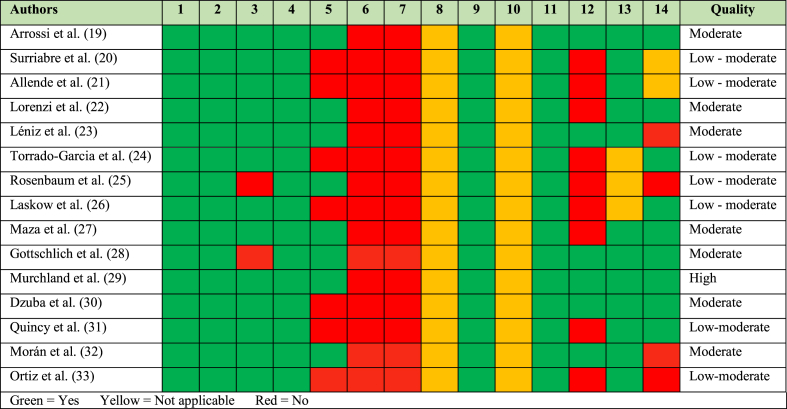
The National Heart, Lung and Blood Institute - based study quality criteria [21]

## 3 Results

### 3.1 Study characteristics

**Fig. 1:**
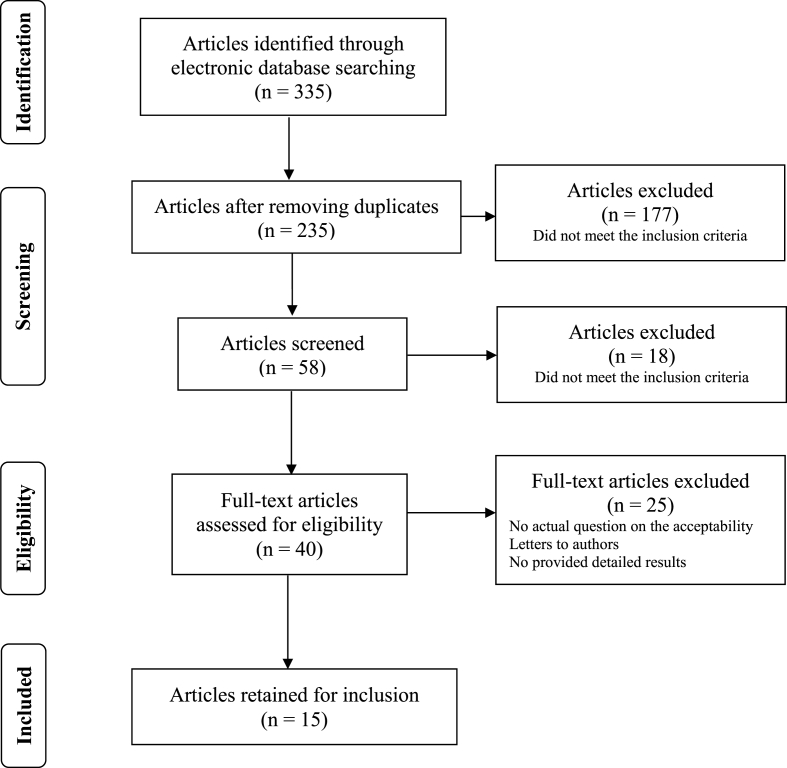
Study selection flow chart.

Fig. 1 shows the selection process for studies included in the review. A total of 335 citations were yielded in the search using the keywords previously described. After removing duplicate reports, a total of 235 articles remained. Through an initial reading of titles and abstracts meeting the inclusion criteria, 58 potential articles of interest were selected. Finally, 40 full-text articles were obtained and read using the same selection criteria; specific articles were selected for further review and final analysis. Of these, 15 articles were included in this review.

Table 2 summarizes the fifteen included articles published between 2002 and 2020 [[22], [23], [24], [25], [26], [27], [28], [29], [30], [31], [32], [33], [34], [35], [36]]. Three of the studies were conducted in El Salvador [[28], [29], [30]], two in Bolivia [23,24] and Guatemala [31,32], and one each in Argentina [22], Brazil [25], Chile [26], Colombia [27], Mexico [33], Nicaragua [34], Peru [35] and Puerto Rico [36]. 60% were conducted in Central America [[28], [29], [30], [31], [32], [33], [34],^36^]. El Salvador and Guatemala have the highest publications, with 20% [[28], [29], [30]] and 13.3% [31,32]. The studies were carried out among women living in urban, peri-urban, and rural areas, the latest the most studied. All the publications were cross-sectional studies, two of which used a mixed methodology [22,35].

**Table 2:** Summary of the fifteen included articles.

Country	Authors, Year	Study design	Women, *n*	Population characteristics	Intervention	Setting	Acceptability	Experience
**Argentina,** Jujuy (Urban and rural area)	Arrossi et al., 2016 [22]	Cross-sectional/Mixed method	3049	**Inclusion criteria:** ***Age:*** 30 years + Living in a home visited by community health workers **Exclusion criteria:** Have a previous HPV DNA test History of hysterectomy History of treatment for premalignant or malignant disease Pregnancy Have a mental disability	Self-sampling **Offered by:** Community Health Workers (CHWs) **Specimen collection instructions:** offered but not described **Device:** cervical sampler kit (Qiagen, Gaithersburg, MD, USA), brush. **Quantitative component** ***Questionnaire:*** 7-item closed-ended questions regarding education level, health insurance, cervical cancer screening history, and reasons for screening method choice. **Qualitative component** Two focus groups (*n* = 30) Interview for HPV knowledge, reasons for accepting or rejecting self-sampling tests. Experience, satisfaction, and circumstances surrounding the test. The possibility of changing their minds in the future to accept self-collection.	Home	85.8% for self-sampling	Majority accepted for being comfortable, easy, fast, painless, voluntary, and free.
**Bolivia,** Cochabamba (Urban, peri-urban, and rural area)	Surriabre et al., 2017 [23]	Cross-sectional	222	**Inclusion criteria:** ***Age:*** 25 -59 years old Living in urban, peri-urban, and rural areas of Cochabamba.	Self-sampling and clinician-sampling. **Offered by:** a health professional **Specimen collection instructions:** written and visual (video) **Device:** cotton swab and vaginal tampon ***Questionnaire:*** evaluate the experience with self-sampling and the preference for a specific device	Health center	64% for self-sampling	Comparing the two self-sampling devices: Cotton swab is 77% easier to use, and 80% more comfortable to use than a vaginal tampon.
**Bolivia,** Cochabamba and Chapare (Urban, peri-urban, and rural area)	Allende et al., 2019 [24]	Cross-sectional	221	**Inclusion criteria:** ***Age:*** 25 -64 years old Living in urban, peri-urban areas of Cochabamba and rural Chapare Signed informed consent **Exclusion criteria:** Pregnant women over 20 weeks History of hysterectomy	Vaginal self-sampling and physician-sampling **Offered by:** a health professional **Specimen collection instructions:** offered but not described **Device:** cotton swab ***Questionnaire:*** 8-item closed-ended questions after self-sampling and physician-sampling	Health center	High acceptance of self-sampling	89.7% easy to use 81.7% comfortable 67.2.% painless
**Brazil,** São Paulo (Urban area)	Lorenzi et al., 2019 [25]	Cross-sectional	116	**Inclusion criteria:** ***Age:*** 21 years + Were referred for colposcopy due to an abnormal Pap smear. **Exclusion criteria:** Women under 21 years of age Pregnant women Women unwilling to participate in the research protocol	Vaginal self-sampling **Offered by:** a health professional **Specimen collection instructions:** verbal and visual (illustrations) **Device:** Evalyn Brush® (Rovers®, Oss, the Netherlands). ***Questionnaire:*** 7-item regarding ease of understanding of the method's use, ease of the use the self-collection brush, discomfort or pain, embarrassment or shame, fear of hurting oneself, preference between self-sampling vs. health professional collection. Reason to choose self-sampling (less pain or discomfort, less shame or embarrassment, practicality, Self-sampling at home/Basic Health Facility/ Laboratory; afraid of not collecting it correctly, the health professional can do it better)	Health center	76.70% for self- sampling (95% CI, 68.40–83.70) vs. 12.9% for health professional sampling (95% CI, 7.8–19.9%) vs. 10.3% for both tests acceptable (95% CI, 5.8–16.9%)	Easy to understand how to use and use it. Practicality, minor embarrassment, Discomfort or pain perception decreased as the age increased (*p* = 0.080).
**Chile,** Santiago (Urban area)	Léniz et al., 2013 [26]	Cross-sectional	1085	**Inclusion criteria:** ***Age:*** 30–64 years Residents of the geographic area covered by the Alejandro del Río health center in the Puente Alto County Have not attended Pap screening in the previous three years. **Exclusion criteria:** History of hysterectomy Pregnant women	Vaginal self-sampling **Offered by:** Community Health monitor **Specimen collection instructions:** verbal **Device:** HC2 Collection Device (brush) ***Questionnaire:*** regarding socio-educational characteristics, reproductive history, Pap test history, smoking, sexual habits, satisfaction with the procedure, and future test preference	Home	High acceptability for self-sampling	93.4% slightly or not at all uncomfortable 91.6% considered vaginal self-sampling less uncomfortable than Pap testing
**Colombia,** Bucaramanga (Urban area)	Torrado-Garcia et al. 2020 [27]	Cross-sectional	423	**Inclusion criteria:** ***Age:*** 35–65 years Living in the northern part of Bucaramanga Have a moderate to high risk of developing cervical cancer **Exclusion criteria:** History of hysterectomy Pregnant women	Cervico-vaginal self-sampling and physician-sampling **Offered by: a** health professional **Specimen collection instructions:** visual and verbal **Device:** brush ***Questionnaire:*** 10 questions regarding experience, comfort, the safety of the procedure, preference between the self-sampling method and conventional cytology, and the reasons why they had chosen one of the two methods	Health center	88.5% for self-collected sampling vs. 4% for conventional cytology vs. 7.3% no preference over any method	40.1% Privacy 29.7% comfortability 14% easier to use 29.7% painless 12.4% reliability
**El Salvador,** San Pedro Perulapan, San Rafael Cedros, Apastepeque and San Sebastian (Rural area)	Rosenbaum et al., 2014 [28]	Cross-sectional	518	**Inclusion criteria:** ***Age:*** 30–49 years Under-screening women in the last 3 years Women capable of providing informed consent **Exclusion criteria:** Pregnant women History of hysterectomy, cryotherapy, or loop electrosurgical excision procedure	Provider-collected sampling and cervicovaginal self-sampling **Specimen collection instructions:** verbal **Offered by:** Health provider **Device:** careHPV QIAGEN Gaithersburg, Gaithersburg, MD, USA) ***Questionnaire***: regarding demographic information (age, education, marital status, household size, and the number of children), sexual history (age of first intercourse, lifetime sexual partners, and current birth control method), smoking history, cervical cancer screening history, and knowledge of HPV and cervical cancer. Open-ended question regarding the preference between self-sampling or provider-collected sampling, preferred method and during a future screening visit, the preferred screening location (home vs. clinic).	Health center	38.8% for self-collection; (95% CI, 34.6–43.2) vs. 31.9% for provider-collected sampling (95% CI, 27.9–36.1) vs. 29.3% no preference over any method. (95% CI, 29.3–33.5)	29.9% Privacy/embarrassment 19.9% ease 18.9% pain 14.9% comfort 8.5% time/convenience
**El Salvador,** San Pedro Perulapan, San Sebastian, Apastepeque, San Rafael Cedros, Candelaria, San Vicente, Tecoluca, and Suchitoto (Rural area)	Laskow et al., 2017 [29]	Cross-sectional	60	**Inclusion criteria:** ***Age:*** 30–59 years Non-attenders women to scheduled appointments for cervical cancer screening of the CAPE program Women capable of providing informed consent **Exclusion criteria:** Pregnant women Women screened within the past 2 years history of hysterectomy, cryotherapy, or loop electrosurgical excision procedure.	Vaginal self-sampling **Specimen collection instructions:** visual and verbal **Offered by:** Health researchers **Device:** Digene Hc2 DNA test, Gaithersburg, MD, USA) (Brush) ***Questionnaire***: regarding sociodemographic characteristics (age, education, marital status, household size, and number of children), sexual history (age at first intercourse, number of lifetime sexual partners, and birth control method), smoking history, previous cervical cancer screening, knowledge and risk perception of HPV and cervical cancer, and reasons for non-attendance, and reasons for agreeing to self-sampling	Home	68% for self-sampling	90% easy process, could be performed at home, save time, little discomfort. and less embarrassment.
**El Salvador,** San Vicente, La Paz, Cabañas, and Cuscatlán (Rural area)	Maza et al., 2018 [30]	Cross-sectional	1869	**Inclusion criteria:** ***Age:*** 30–59 years Underscreening women (No cytology screening in the last three years, HPV screening within the last five years or had never been screened) **Exclusion criteria:** History of hysterectomy, cryotherapy, cold knife conization History of cervical cancer	Vaginal self-sampling **Specimen collection instructions:** visual and verbal **Offered by:** Community Health promoter and research assistant. **Device:** CareHPV test (QIAGEN, Gaithersburg, MD, USA) ***Questionnaire***: collected sociodemographic information, health, sexual history, previous screening history, cervical cancer and HPV risk perception, and reasons for non-participation in previous screening programs. Finally, separate sets of questions were administered to women who accepted and those who declined self-sampling to explore the underlying reasons.	Home	99.8% for self-sampling	Most women agreed with statements highlighting positive aspects of the test (e.g., it is easy to perform, can be performed at home, and is more comfortable to do the exam oneself).
**Guatemala,** Santiago Atitlán, (Rural and rural area)	Gottschlich et al., 2017 [31]	Cross-sectional	178	**Inclusion criteria:** ***Age:*** 25–54 years **Exclusion criteria:** Pregnant women Women currently menstruating	Cervical self-sampling **Specimen collection instructions:** visual and verbal **Offered by:** Community Health Workers (CHWs): Tz’utujil language **Device:** Eve Medical HerSwab self-collection HPV kits ***Questionnaire***: 143 questions regarding demographics, preventive health care practices, HPV and cervical cancer knowledge, and risk factors. Finally, questions assessing the acceptability and feelings toward HPV self-collection.	Home	High acceptability for self-sampling	78.7% comfortable to use 91% easy to use 80% screening at home
**Guatemala,** Santiago Atitlán, and Livingston (Rural area)	Murchland et al., 2019 [32]	Cross-sectional	760	**Inclusion criteria:** ***Age:*** 25–54 years **Exclusion criteria:** History of hysterectomy, History of previous cervical cancer Pregnant women Women currently menstruating Women who had never been sexually active.	Cervical self-sampling **Specimen collection instructions:** visual and verbal **Offered by:** Community Health Workers (CHWs) (bilingual: Spanish and Tz’utujil or Q’eqchi, Karif language) **Device:** HerSwab kits (brush) ***Questionnaire***: 153 questions regarding demographics, risk factors for cervical cancer and HPV, self-reported attitudes towards screening, health care service use, and knowledge of cervical cancer and HPV. Finally, a post-sample survey of 3 questions regarding ease, comfort, and acceptability of the sampling method:	Home	High acceptability for self-sampling	82.3% comfortable 84% easy to use 96.7% willing to use it as a form of cervical cancer screening
**Mexico,** Morelos (Unspecified area)	Dzuba et al., 2002 [33]	Cross-sectional	1061	**Inclusion criteria:** ***Age:*** 20 years + Use of the Mexican Institute of Social Security services in Morelos Are registered in the parent study [50]	Vaginal self-sampling and health professional sampling **Specimen collection instructions:** visual, written, and verbal **Offered by:** Female nurses (self-sampling and pelvic examination) **Device:** Cotton-tipped sterile Dacron swab ***Questionnaire***: 65 questions regarding socioeconomic and demographic status; sexual, reproductive, and Pap histories; and the acceptability (discomfort, pain, embarrassment, and privacy) perceived during the self-sampling and Pap test procedure.	Health center	65.6% for self-sampling vs. 11.3% for Pap test vs. 23% for both procedures Overall self-sampling acceptability score was 21.7 (*p* < 0.001) for a maximum total score of 25.	71% more comfortable 55.3% less embarrassing
**Nicaragua,** Leon (Unspecified area)	Quincy et al., 2012 [34]	Cross-sectional	245	**Inclusion criteria:** ***Age:*** 25–60 years Women living in Leon, Nicaragua Women with intact uteri **Exclusion criteria** Pregnant women	Vaginal self-sampling and clinician-collected specimen **Specimen collection instructions:** none reported **Offered by: a** health professional **Device:** vaginal swab and brush ***Questionnaire***: questions regarding demographic information, past medical and reproductive history, and perceptions of experiences with self-collection and the clinician examinations. The questionnaire included items about the comfort, pain, privacy, and level of embarrassment associated with the self-collection and pelvic examination. There were also questions about the preference of testing method, the reason for the preference and willingness to self-collect in the future.	Health center	High acceptance of self-sampling Self-collected brush acceptability Score index for a maximum total score of 20 Self-collected brush (M = 18.40, SD = 2.73) Self-collected swab (*M* = 18.48, *SD* = 2.41), *t*(238) = 4.27, *p* < 0.01. Clinician-collection (*M =* 17.56, *SD =* 2.92), *t* (235) = 3.81, *p* < 0.01	76.3% no pain with self-sampling using the swab 73.1% no pain with self-sampling using the brush 76.3% very comfortable with self-sampling using the swab 73.1% very comfortable with self-sampling using the brush 90.2% no embarrassment with self-sampling using the swab 88.2% no embarrassment with self-sampling using the brush 90% high privacy for all methods self-sampling using brush and swab were statistically significantly high than those for the clinician-collection
**Peru,** Ventanilla (Unspecified area)	Morán et al., 2017 [35]	Cross-sectional / Mixed method	97	**Inclusion criteria:** ***Age:*** 25–59 years Have performed a previous vaginal self-sampling test at the HOPE program (Women who help women to fight cervical cancer)	**Previous vaginal self-sampling** **Offered by:** Community Health Workers (CHWs) **Device:***Care*HPV (QIAGEN, Gaithersburg, MD, USA) ***Questionnaire***: 29 questions regarding sociodemographic information and variables of preferences regarding self-administration of the test.	Home	68% for self-sampling	It requires less time, privacy. very few women reporting pain or discomfort.
**Puerto Rico,** San Juan (Urban area)	Ortiz et al., 2012 [36]	Cross-sectional	100	**Inclusion criteria:** ***Age:*** 18–34 years Women undergoing routine Pap smears in the University of Puerto Rico Gynecology Clinic. Women with an intact uterus, No history of cervical cancer No recent cervical procedures **Exclusion criteria:** HIV-positive Cognitively or physically impaired	Cervicovaginal clinician-collected specimens and cervicovaginal self-sampling **Specimen collection instructions:** written and verbal **Offered by:** physician **Device: Sterile collection kit -** Dacron swab and Cytobrush® (Cooper Surgical, Inc; Connecticut, USA) ***Questionnaire***: 16-item questions regarding demographic, lifestyle, and reproductive characteristics. Sexual practices and acceptability (comfort, pain, privacy, and embarrassment) and the reasons for this preference for the self-sampling for HPV testing	Health center	50% for self-sampling. vs. 22% for clinician-collection vs. 28% for both sampling methods (MD = −0.71, p<0.05). MD: mean difference	Less embarrassment (MD − 0.36) Less pain (MD − 0.23) Women felt that the techniques were equally acceptable in terms of pain (58%), embarrassment (71%), discomfort (47%), and privacy (94%).

A total of 10,004 women who performed the HPV self-testing were surveyed. Regarding inclusion criteria, the enrolled women were 18 years or older; however, most studies screened women with an average of 25 to 59 years of age for screening with the self-testing method; the selected age range varied according to the characteristics of each study and guidelines for cervical cancer prevention in each country. Several studies focused on including under-screened women within the previous three years at the time of the study and non-attendees to cervical cancer screening appointments fixed by local prevention programs. Only one study focused on women having a moderate to high risk of developing cervical cancer [27]. The studies conducted in Guatemala focused on indigenous women [31,32]. The most frequently employed exclusion criteria were being pregnant, having a history of cervical cancer, undergoing a hysterectomy, or receiving other treatments for cervical abnormalities. The primary language of the studies was Spanish; the indigenous communities spoke Tz'utujil, Q'eqchi, or Karif. Therefore, bilingual community health workers assisted them in facilitating their understanding of the information provided during the studies.

The proportion of studies that evaluated the self-sampling method alone was 53.3%, compared to 46.7% of the remaining publications, which used both methods (self-sampling and health professional sampling). Health professionals offered for 60% of the HPV self-tests, as they also conducted cervical cytology in certain cases, whereas the remaining 40% were offered by Community Health Workers (CHWs). Similar rates were observed with regard to the choice of screening setting (health center 53.3% vs. home 46.7%).

Concerning the type of device tested, various brands were used. Up to 53.3% of women were offered to use a brush as a collector, and 26.7% opted to use the swab. Two studies employed both brushes and swabs simultaneously [34,36]; a single study incorporated the vaginal tampon and swab in its evaluation [23]. The self-sampling instructions were given both verbally and visually (at the same time) in 40% of cases, the instructions were only given verbally in 13.3% of cases, a combination of both written and visual, written and verbal, or oral, written, and visual instructions were reported in 6.7% of cases, respectively. The remaining studies did not describe the instructions for the self-sampling method. After performing the test, all studies were based on non-standardized questionnaires with heterogeneous questions to assess the acceptability of the HPV self-sampling test among women. Two studies relied additionally on a qualitative component with focus groups and guided interviews [22,35].

Eight papers reported a high acceptance of HPV self-sampling. Among these, three papers reported an acceptance of the self-testing greater than 80% [22,27,30], of which Maza et al. [30] reported near 100% acceptability. Additionally, the other five publications reported the acceptance of HPV self-sampling as “high” without specifying it directly in terms of percentages [24,26,31,32,34]. Six papers found an acceptance level of between 50 and 80% [23,25,29,33,35,36]. Only one study found an acceptance level of lower than 50% [28]. Five publications examined the acceptability of self-sampling vs. provider sampling collection vs. the two methods simultaneously; among which Torrado-Garcia et al. [27], Lorenzi et al. [25], and Dzuba et al. [33] evidenced self-sampling acceptability rates of 88.5%, 76.7%, and 65.6% respectively over the health professional collection or the two methods equally. Rosenbaum et al. [28] and Ortiz et al. [36] indicated a self-sampling acceptance of 38.8% and 50%, respectively. Although acceptance was lower than 50%, there is a greater proportion of women who conduct self-testing than those who choose the physician-collection method or use a combination of both approaches. The results of Rosenbaum et al. [28] tend towards similar acceptance rates among those who select self-sampling (38.8%), samples collected by a health professional (31.9%), and those who accept both methods (29.3%). Dzuba et al. [33] show that in spite of the high acceptance of self-testing, 23% of women accept both methods equally, compared to 11.3% who accept only the test de Papanicolaou (Pap test) a Physician-collected method.

The leading indicators for assessing the acceptability of each method were comfort, ease of use, pain, embarrassment, and privacy. Around 66% of the women considered self-testing to be a comfortable method when compared to the physician-collected method [23,24,[26], [27], [28],[31], [32], [33], [34],^36^], and referred to it as an easy [[22], [23], [24], [25],[27], [28], [29], [30], [31], [32]] method. In terms of pain, roughly 50% of the women considered it less painful [22,24,27,28,[34], [35], [36]] than conventional physician-collected method, they also felt less embarrassed [28,29,33,34,36], and felt it gave them more privacy [27,28,[34], [35], [36]] than the routine procedure, involving a gynecological examination. Among these indicators, Quincy et al. [34] found that comfort, pain, and embarrassment showed a statistically significant (*p* < 0.001) predictive relationship for the uptake of vaginal self-testing. However, other indicators analyzed more infrequently were screening rapidity [22,28,29,35], ease of understanding screening instructions [25,27], fear of hurting themselves [25], the willingness to undertake self-testing as a screening method in the future [28,[30], [31], [32], [33]], and the possibility of undertaking self-testing at home [[28], [29], [30], [31]]. Nevertheless, Laskow et al. [29] and Maza et al. [30] used women’s Likert scores to evaluate the experience with vaginal self-testing, and they found that women reported being highly satisfied with the experience, with an average of 9.5 on a 10-point scale and between 4.2 and 4.6 on a 5-point scale, respectively.

Furthermore, various studies tested different devices, such as swabs, brushes, and tampons; the results showed that women thought that the swab was more comfortable (80% vs. 56%) and easier to use (77% vs. 59) than the vaginal tampon [23]. Regarding the acceptance of self-testing performed with a swab or a brush, Quincy et al. [34] revealed a slightly higher acceptability of the swab (*M =* 18.48, *SD =* 2.41) over the brush (*M* = 18.40, *SD* = 2.73) when measuring the acceptability index over a maximum score of 20 points.

### 3.2 Reasons limiting the acceptance of cervical cancer screening

Table 3. Summarizes the reasons that may limit the acceptance of HPV self-sampling or other cervical screening methods. The primary reasons for not accepting the HPV self-sampling screening test were related to beliefs about the inaccuracy of its results [28,29,34,35], concerns over the ability to correctly use the self-sampling tool [22,24,30], beliefs about the possibility of getting hurt [22,25,30], or concerns about feeling discomfort or pain during self-sampling [25,28,30], a lack of interest in one’s own health [22,26,29], and the perception of not having the disease, due to the absence of self-observed symptoms [22,29,30]. Other reasons, which were not often mentioned, were associated with the perception of, and beliefs about the self-sampling test, including; the fear of contaminating the sample [22,28,36], the possibility of having a cervical cancer diagnosis [22,35], the lack of confidentiality in healthcare facilities [22,30], the belief that cancer is a dormant disease that can be provoked by introducing a sample-taking device in the vagina or cervix [22]. Elsewhere, studies have revealed that women may refuse the self-sample test because they prefer attending a health center [26,29], can be embarrassed by self-sampling, and feel uncomfortable touching themselves. In addition, some women have reported having greater confidence in the Pap test [33] and the knowledge and expertise of the test provider [28]. Furthermore, Maza et al. [30] reported that women may not attend screening appointments because they feel embarrassed to be examined by a male physician, believe that cervical cancer screening is unnecessary, and fear that treatment would be needed.

**Table 3:** Reasons limiting the acceptance of HPV self-sampling.

Country, Authors	Reasons to not accept HPV self-sampling or other screening test
**Argentina,** Arrossi et al. [22]	Insecurity in their ability to correctly use the self-sampling test
	Possibility of self-injury using the self-sampling test
	Fear of contaminating the sample
	Lack of confidentiality in healthcare facilities
	Perception of the health-disease status defined as the absence or presence of symptoms (pain, inflammation, or vaginal discharge)
	Lack of interest in their health
	The possibility that screening could result in cancer diagnosis frightened women
	The belief that cancer is a dormant disease that can be awaken by introducing a sample-taking device in the vagina or cervix
**Bolivia,** Surriabre et al. [23]	NR
**Bolivia,** Allende et al. [24]	NR
**Brazil,** Lorenzi et al. [25]	Fear of self-injury using the self-sampling test
	Discomfort or pain using the self-sampling test
**Chile,** Léniz et al. [26]	Lack of interest (38.2%)
	Preference to attend health center (26.5%)
	Fear of the procedure (19.6%)
	Lack of time (15.7%)
**Colombia,** Torrado-Garcia et al. [27]	NR
**El Salvador,** Rosenbaum et al. [28]	Result accuracy (33.3%)
	Provider’s knowledge confidence (24.2%)
	Confidence in the provider's expertise in performing the test (16.4%)
	Fear of improper sampling (13.3%)
	Comfort (33.0%)
	The availability of assistance/equipment (25.2%)
	The sanitation of the facilities (12.4%)
	Privacy (11.0%)
**El Salvador,** Laskow et al. [29]	Disinterest to be screened (*p* = 0.001)
	Belief that the results might not be correct
	Discomfort with touching themselves (*p* = 0.001)
	Felt embarrassed by self-sampling (*p* = 0.001)
	Preferred that a clinician take the sample (*p* = 0.001)
	Not having the time or privacy in their own home (*p* = 0.001)
	Perception to be at low risk of cervical cancer to not have symptoms
**El Salvador,** Maza et al. [30]	Were embarrassed at being seen by a male physician (55.6%) *
	Lack of symptoms (38.9%) *
	Belief that the test was not necessary (27.5%) *
	Long clinic waits times (22.5%) *
	Belief that the screening would be painful (27.1%) *
	Fear that treatment would be needed (20.5%) *
	Belief that tests results would not be kept confidential (20.1%) *
	Fear that the person might lose part of the uterus during treatment (22.9%) *
**Guatemala,** Gottschlich et al. [31]	NR
**Guatemala,** Murchland et al. [32]	NR
**Mexico,** Dzuba et al. [33]	More confidence in the Pap test (93.1%)
**Nicaragua,** Quincy et al. [34]	More confident of the result from clinician-sampling
**Peru,** Morán et al. [35]	Fear of knowing they are diseased
	Confidence that self-sampling will be administered correctly
	Distrust in the validity of self-sampling results
**Puerto Rico,** Ortiz et al. [36]	More confident that the sample would be more properly taken (85.6%)

**NR:** Not reported.

* Reasons for not attending a cervical cancer screening appointment.

## 4 Discussion

Cervical cancer continues to be a significant public health problem affecting women worldwide [[3], [4], [5]]. The effort to detect women at high risk of cervical cancer is challenging as there are several difficulties establishing a screening programme and access to ad hoc cervical testing in LMICs. This is the first review to summarize the acceptance of HPV vaginal self-sampling as a screening method for cervical cancer in Latin America [[22], [23], [24], [25], [26], [27], [28], [29], [30], [31], [32], [33], [34], [35], [36]].

The different research studies included in this review have demonstrated high acceptability of HPV self-sampling compared to the clinician-collection, despite the different devices used; these results are consistent with Nodjikouambaye et al. [37]studies previously conducted in the African context. Even though the acceptance of this methodology in Latin America was subjective because no standardized questionnaire for evaluating vaginal self-sampling acceptability exists, the authors demonstrated the common points of acceptance and the facility of approaching women who had not wanted to be screened by conventional physician-collected method. However, women highlighted the simplicity of vaginal self-sampling because of the limited time required to perform it and the possibility of performing it at home [22,25,[28], [29], [30],^35^].

In addition, the Community Health Workers (CHWs) support played an essential role in the approach of the women who have impairments in approaching health centers provided for the traditional cervical cancer screening tests, and also when women have linguistic communication barriers because they speak regional dialects and do not speak Spanish [22,26,[30], [31], [32],^35^]. The literature demonstrates that offering HVP testing through CHWs during home visits effectively increases cervical cancer screening coverage [38,39].

Nevertheless, this systematic review highlighted some reasons that can limit the acceptance of the self-sampling approach; mainly women's perceptions of and beliefs about this screening method. The most outstanding reason was related to beliefs about the accuracy of results. HPV self-sampling has no statistically different specificity or sensitivity compared with clinician-collection samples [40]. Some studies have previously demonstrated a high level of agreement for the detection of HPV DNA through self-sampling testing and clinician-collection samples [41,42]. These results are similar to those reported by Surriabre et al. [23] in Bolivia and Torrado-Garcia et al. [27] in Colombia, where similar identification levels were found for the two screening methods with a Cohen’s Kappa = 0.71 (95% CI 0.55 -0.88) and 0.9774, respectively.

Despite the cultural barriers mentioned above, there are other barriers such as religious, socioeconomic, demographic, and geographical factors. However, none of these other barriers were identified in the current review, probably due to the structure of the questionnaires used in the selected articles. A previous study by Allen-Leigh et al. [43] revealed a series of barriers to cervical cancer screening among indigenous Mexican women in rural communities; these barriers related to the lack of knowledge about self-sampling and human papillomavirus, male partner opposition, and organizational barriers such as the distance to the clinic, use of comprehensive language and long waiting times in receiving test results. In addition, this study also refers to the lack of confidence to perform the self-sampling procedure correctly, in keeping with concerns revealed by our systematic review.

Finally, women consider some reasons such as the reliability of the sample taken, the experience or performance of the clinician, fear of injury, discomfort of touching their own vulva, and hygiene at the time of screening, to be particularly important. Such concerns highlight the need for a clinician-collection sampling option, alongside that of self-sampling [28,29,35,36].

The HPV self-sampling procedure is a useful solution to reduce barriers to access for screening programs in LMICs. Moreover, increasing the use of self-sampling methods in LMICs may help ration the more resource intensive physician-collected methods to those women who are determined to be higher risk based on the self-sampling results. In Bolivia - one of the countries with the highest incidence and mortality rates of cervical cancer in Latin America - Allende et al. observed a 4.7 fold increase in self-sampling by women living in a peri-urban area, when compared to rates from the previous year [24]. These data were similar to others obtained in Argentina, Guatemala, and Mexico, where a 4-fold increase in self-sampling screening coverage was observed [31,38,43]. In addition, HPV self-sampling is a cost-effective screening method, especially in LMICs, compared to an average cost of a Physician collected method in Latin America, it can range from 13.00 to 72.00 US Dollars (USD) on average [44]. Particularly, Surriabre et al. [23], reported that an HPV self-sampling kit containing a pair of sterile gloves, a cotton swab, and a sterile glass slide covered in a cardboard box could have an accessible price of around 0.5 USD. Other results were obtained recently in Peru in a real-world implementation of HPV self-sampling; the cost per HPV self-sampling kit distributed door-to-door by community health workers to women with a socioeconomic disadvantage for around 3.00 USD, this same kit could be obtained and tested for women of higher socioeconomic status for around 45·39 USD [45,46]. Self-sampling therefore costs less than cytological screening and treating a premalignant lesion or the same cervical cancer [46].

In May 2018, a global call for action to eliminate cervical cancer was announced by the Director-General of the World Health Organization (WHO) [47]; in August 2020, The World Health Assembly adopted the Global Strategy to eliminate this disease, in which they pointed out that providing women with the option of self-sampling contributes to acceptability and access to health services [15]. According to the facts mentioned above and to the experience gained after the effects of the COVID-19 pandemic, where traditional cervical cancer screening programs were disrupted, the self-screening initiatives can change the perspectives of vaginal self-sampling as a screening method for women worldwide [48,49].

This review has some limitations that need to be addressed. 1) The heterogeneity of the populations in terms of demographic data and sample size varied among the studies. It can be explained by the vast age range of the participants included in each study, the number of patients ranging from 60 to 3049, and the difficulty of generalizing the results to some populations, such as indigenous communities. 2) No standardized questionnaire was used. Therefore, the variety of questions in the studies make it difficult to compare them. In a few studies, the authors asked about the reasons for refusing self-sampling, the possibility of changing their minds about this screening method, and their cervical cancer knowledge. For this reason, it is necessary to create a standardized and validated questionnaire to carry out further research and obtain more accurate results on the acceptability of vaginal self-sampling by women in the Latin American context. 3) Most publications were carried out in El Salvador, Guatemala, and Nicaragua - three out of the six Latin American countries located in Central American countries - with one of the highest prevalence of cervical cancer in Latin America. In this region, different investigations have been carried out to introduce HPV testing for cervical cancer, such as the Scale-Up project in Guatemala, Honduras, and Nicaragua [50]; and the CAPE project (The Cervical Cancer Prevention in El Salvador) for El Salvador [51]. We note that the acceptability of HPV self-sampling was not investigated at the time these projects were introduced. Nevertheless, evaluation of the Scale-Up project has shown a positive impact, with an 85.5% target coverage of HPV-based screening, where 75,1% of the total women screened for HPV used the self-sampling collection. In this regard, it is also essential to conduct future studies that explore the acceptance of vaginal self-sampling in other countries and settings with greater inclusion of indigenous groups and women with HIV infection. 4). Finally, the fact that only one researcher has performed the quality analysis of the articles may lead to selection and information bias. Finally, certain studies mixed up the terminology of acceptability with test preference, making it difficult to interpret the results in some cases.

## 5 Conclusion

Vaginal HPV self-sampling is an additional screening method, a helpful cost-effective tool [52] with a high acceptance among Latin American women that can achieve a higher rate of screening vulnerable populations and ethnic minorities at risk of cervical cancer. This strategy increases women's opportunities to participate actively in cervical cancer prevention and increases screening coverage and greater adherence to screening programs. In addition, early diagnosis of the disease will allow timely initiation of treatment and follow-up care.

Considering that self-sampling is an effective screening method that provides privacy, autonomy, and confidentiality, it is essential to continue the education campaigns about the existence of the human papillomavirus, its relationship with cervical cancer, the possibility and advantage of vaccination for HPV, the methods and frequency of the screening test, in the aim of e reducing cervical cancer’s incidence and related deaths worldwide.

## Ethics approval and consent to participate

This article does not require ethical approval for being based on previously published data.

## Data availability statement

The authors will make available data upon a reasonable request.

## Declaration of competing interest

The authors declare that they have no known competing financial interests or personal relationships that could have appeared to influence the work reported in this paper.
